# Near-Infrared Fluorescence Imaging for Sentinel Lymph Node Identification in Melanoma Surgery

**DOI:** 10.7759/cureus.14550

**Published:** 2021-04-18

**Authors:** Francisco Ferri, Lisandro Montorfano, Stephen J Bordes, Craig Forleiter, Martin I Newman

**Affiliations:** 1 General Surgery, Cleveland Clinic Florida, Weston, USA; 2 Surgical Anatomy, Tulane University School of Medicine, New Orleans, USA; 3 Plastic and Reconstructive Surgery, Cleveland Clinic Florida, Weston, USA

**Keywords:** near-infrared fluorescence imaging, melanoma, melanoma surgery, sentinel lymph node (sln)

## Abstract

Although less common than other types of skin cancers, melanoma is accountable for the majority of skin cancer-related deaths. The standard management for patients with clinically negative nodes includes a sentinel lymph node (SLN) biopsy, which is commonly performed using a combination of radioactive tracer (Tc-99) and a blue dye (isosulfan or patent blue). There are numerous drawbacks associated with Tc-99 and blue dyes such as elevated costs, logistical challenges, and anaphylactic reactions among others. In recent years, near-infrared (NIR) fluorescence imaging using indocyanine green (ICG) has emerged as a safe, effective, less costly, and more convenient alternative for the identification of SLNs in melanoma. We discuss the case of a 51-year-old man with melanoma in his left upper back. Two SLNs in the left axilla were successfully identified using NIR fluorescence. NIR fluorescence with ICG for SLN identification has proven to increase the sensitivity and accuracy when used in combination with lymphoscintigraphy.

## Introduction

Melanoma represents only 3% of the skin cancers diagnosed each year; however, the disease is accountable for approximately 65% of skin cancer-related deaths [1]. Standard management for patients with clinically node-negative melanoma includes evaluation of regional lymph nodes by means of a sentinel lymph node (SLN) biopsy [2]. SLN biopsy is classically performed using a blue dye (isosulfan or patent blue) and a radioactive tracer (Technetium [Tc-99]) in order to identify the first lymph node or nodes that drain the primary melanoma site [2]. Despite advances in medical technology, pathologic analysis, and surgical techniques, false-negative SLN biopsy is still reported to occur in 13% of cases [3]. Furthermore, both Tc-99 and blue dyes have drawbacks such as possible supply shortages, logistical challenges between the operating room and radiology, staining in surrounding tissues, increased infectious complications, and potential skin necrosis [4]. Recently, near‐infrared (NIR) fluorescence imaging using indocyanine green (ICG) has emerged as an alternative method of SLN identification [5]. ICG is a water-soluble tricarbocyanine dye, which enables deep penetration into tissues, low light scattering, and tissue autofluorescence [6-8]. The use of ICG is relatively safe and non-toxic since the standard dose (2 mg/kg) is significantly lower than the lethal dose (80 mg/kg), with the exception of iodide reactions which are uncommon [6,8]. ICG offers potential advantages to radiotracer-based lymphoscintigraphy including an excellent safety profile, good tissue penetration, and real-time intraoperative imaging capabilities [5,8]. We discuss a case of melanoma for which two SLNs were identified using ICG NIR fluorescence imaging during surgical excision.

## Case presentation

A 51-year-old man with end-stage renal disease was referred to a dermatologist for full-body skin evaluation as part of a kidney transplant preoperative workup. The patient noted brown-colored nevi all over the body but none of them had acutely changed, itched, or bled. During the initial visit, three shave biopsies were taken from suspicious, discolored lesions on the left upper back and right shoulder. The biopsy from the left upper back was positive for a 2.9-mm thick melanoma with a mitotic rate >1/mm^2^ and no ulceration. The lesion involved the deep and peripheral margins. The remaining two biopsies were positive for basal cell carcinoma. The patient was referred to the plastic surgery service for further management of melanoma.

**Figure 1:**
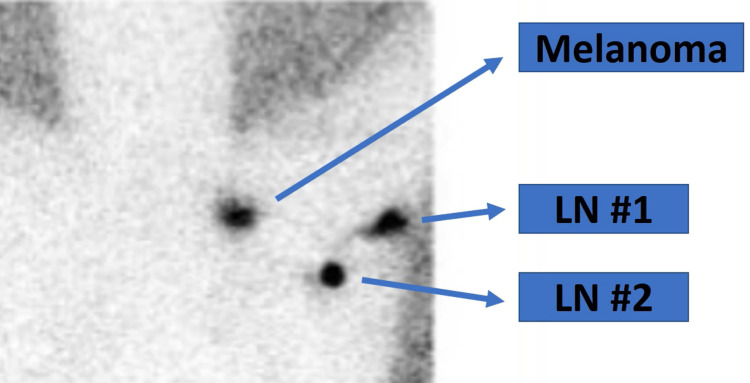
Preoperative lymphoscintigram LN: lymph node

**Figure 2:**
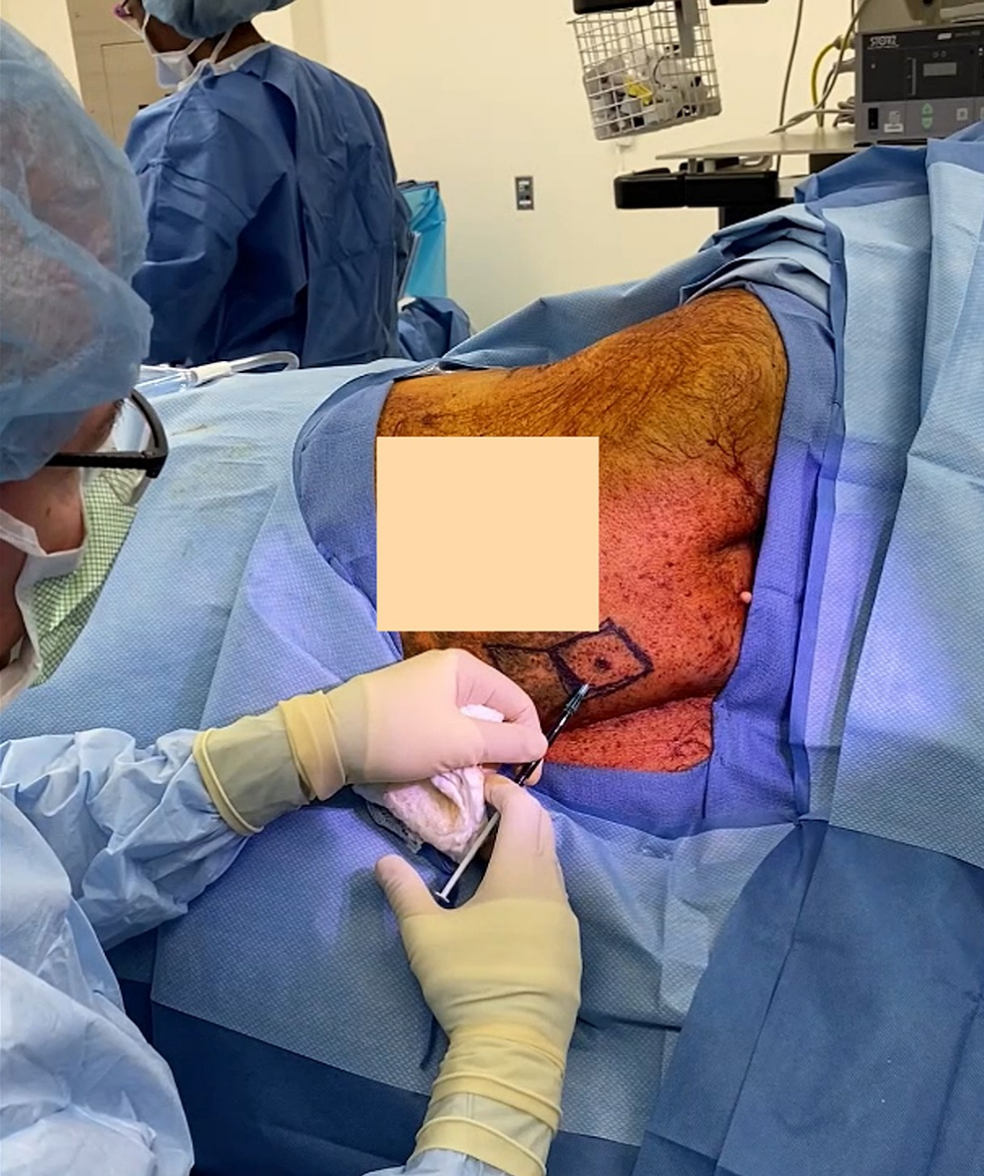
Peritumoral injection of indocyanine green

**Figure 3:**
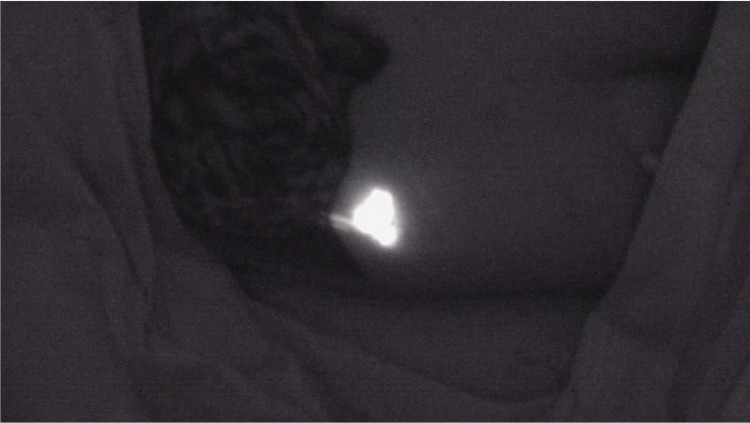
Visualization of tumor under near-infrared light

**Figure 4:**
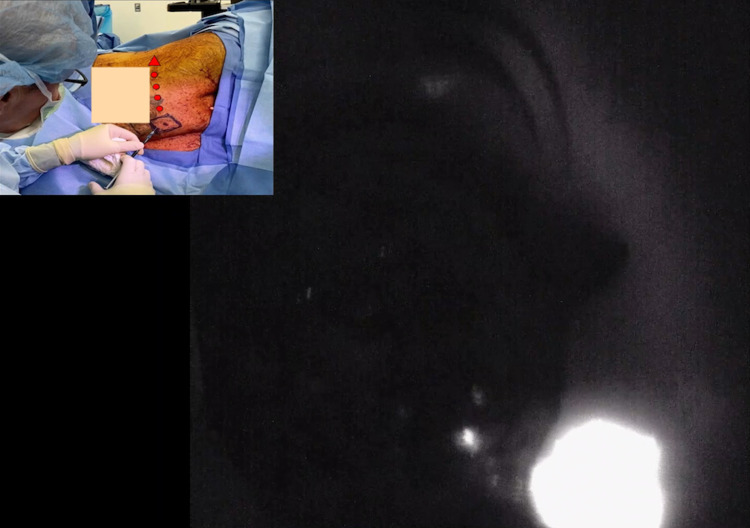
Lymphatic mapping under near-infrared light

**Figure 5:**
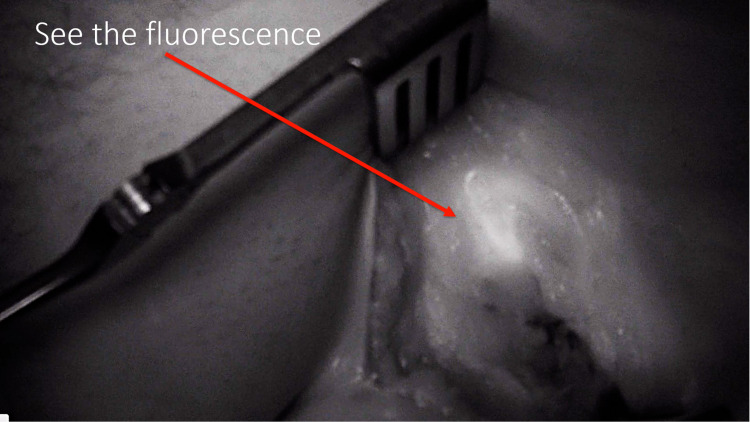
Identification of sentinel lymph node under near-infrared light

**Figure 6:**
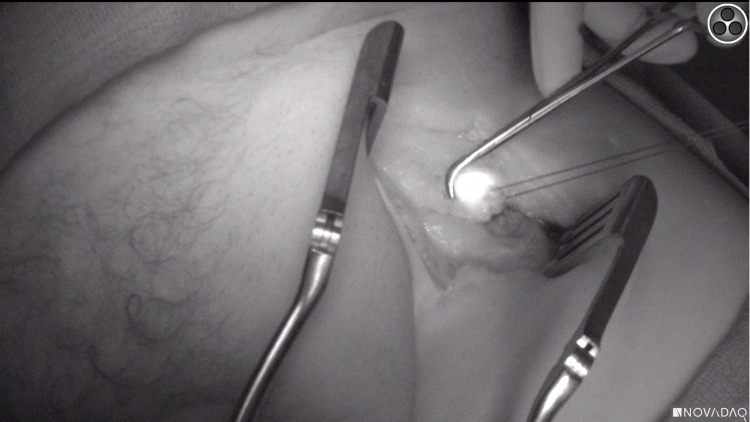
Dissection and excision of sentinel lymph node under near-infrared light

**Figure 7:**
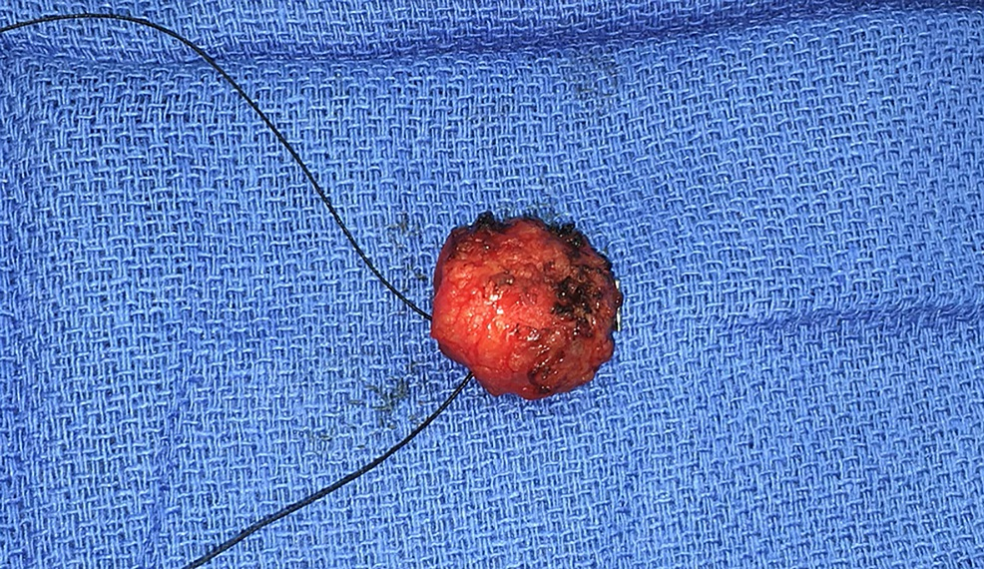
Sentinel lymph node ex-vivo under white light

**Figure 8:**
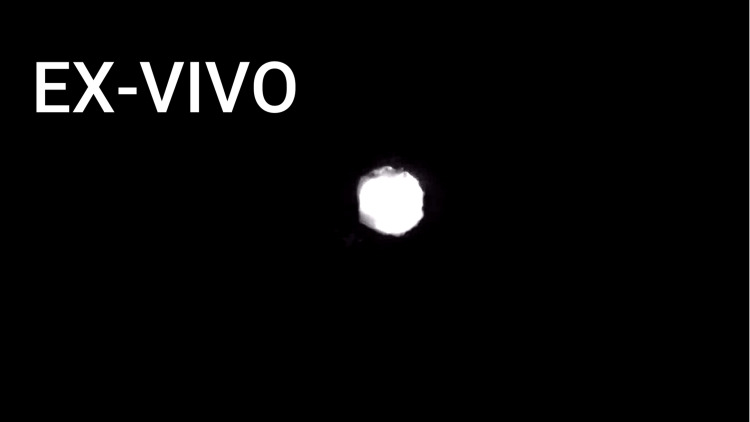
Sentinel lymph node ex-vivo under near-infrared light

Preoperatively, a positron emission topography (PET) scan showed no evidence of metastatic disease. The patient was given a preoperative injection of Tc-99 on the morning of surgery (Figure 1). Under general anesthesia, 0.1 mL of ICG was injected intradermally into the melanoma (Figure 2). A wide local excision of the melanoma was performed with cold knife in a rhomboid fashion. Our attention then turned to the left axilla. A gamma probe confirmed the presence of two SLNs as revealed by lymphoscintigram. An NIR light subsequently identified two hotspots on the skin, which corresponded to the two SLNs. We were able to identify the tract from the intra-lesion injection of ICG toward the left axilla and into two SLNs (Figures 3-6). These intraoperative findings were consistent with the gamma probe and preoperative lymphoscintigram findings. The SLNs were excised and sent to the pathologist (Figures 7, 8).

The patient was discharged home later that day. His postoperative course was uneventful. Final pathology results revealed residual invasive malignant melanoma (Breslow thickness 1.5 mm) with negative deep (1.3 cm) and peripheral (2.5 cm) margins. Both SLNs were benign.

## Discussion

Although lymphoscintigraphy remains the gold standard in the identification of SLNs for melanoma, the process is costly, time-consuming, and logistically challenging [9]. Conversely, the use of ICG has emerged as a powerful tool in the assessment of SLNs in cases of melanoma due to its effectiveness, favorable safety profile, low cost, and convenient application [9].

A recent systematic review of 1,209 patients with cutaneous melanoma reported an SLN detection rate of 86%-100% when using NIR fluorescence with ICG, 86%-100% when using radiotracer, and 43%-73% when using blue dye [10]. Additionally, 2% of nodes were detected only by ICG and not by any other technique. The rate of missed nodes with ICG was heterogenous, ranging from 0% to 13.7% [10]. All of the studies used ICG in addition to preoperative lymphoscintigraphy, as we did. The authors concluded that NIR fluorescence may be a useful adjunct to lymphoscintigraphy in SLN biopsy for melanoma care.

Similarly, a prospective trial analyzed 574 patients who underwent lymphoscintigraphy and NIR fluorescence with ICG for SLN detection in primary cutaneous melanoma [11]. The authors found that 85.3% of SLNs were detected by a combination of both techniques, 14% by lymphoscintigraphy only, and 0.9% by ICG only. Additionally, the SLN positivity rate (21.4%) was higher than the expected rate based on previous publications [11]. This higher SLN positivity rate will theoretically reduce the future rate of false negatives.

It is important to note that the aforementioned publications evaluated the use of NIR fluorescence using ICG in combination with lymphoscintigraphy for primary cutaneous melanoma. A recent systematic review demonstrated that NIR fluorescence with ICG when used in combination with blue dyes or a radiotracer increases node detection sensitivity and accuracy. However, no study recommended the use of ICG as a single agent for SLN mapping in cutaneous melanoma [12].

NIR fluorescence using ICG for SLN detection is a relatively recent technique that has shown to bring significant benefits and improvements to node detection. For certain types of cancer (i.e. breast cancer), it has been suggested that NIR fluorescence could be used alone to reliably perform an SLN biopsy [12]. Regarding primary cutaneous melanoma, larger prospective trials are needed to evaluate this technique as a single agent for SLN identification.

The use of NIR fluorescence with ICG still has its own shortcomings and challenges to overcome. There is significant heterogeneity among studies regarding ICG preparation and imaging protocol [10]. This introduces biases that may affect the results of individual studies. Further studies are required to standardize the technique, which will increase the reproducibility of results. Finally, the use of NIR fluorescence requires technological capabilities that are not readily available in all hospitals throughout the country.

## Conclusions

SLN identification for cutaneous melanoma using NIR fluorescence with ICG is safe and effective. NIR fluorescence with ICG increases the sensitivity and accuracy of SLN detection. Further randomized trials are required to confirm its efficacy as both a single and combined agent for SLN detection in cases of primary cutaneous melanoma.
